# Supplementary material for: HIVprotI: an integrated web based platform for prediction and design of HIV proteins inhibitors
Source: J Cheminform. 2018 Mar 9;10:12. doi: 10.1186/s13321-018-0266-y (PMC5845081; doi:10.1186/s13321-018-0266-y)
Supplement: Supplementary file 2 — Additional file 2. Source code of HIVProtI web server. [file 13321_2018_266_MOESM2_ESM.zip › HIVprotI_Source-code/hivprotI/weka-3-6-6/doc/weka/core/converters/ConverterUtils.DataSource.html]

ConverterUtils.DataSource


---


|  |  |  |
| --- | --- | --- |
| **Overview** \| **Package** \| **Class** \| **Tree** \| **Deprecated** \| **Index** \| **Help** \| **Weka's home** | |  |
| **PREV CLASS**   **NEXT CLASS** | **FRAMES**    **NO FRAMES**     **All Classes** |  |
| SUMMARY: NESTED \| FIELD \| CONSTR \| METHOD | DETAIL: FIELD \| CONSTR \| METHOD |  |


---


## weka.core.converters Class ConverterUtils.DataSource

```
java.lang.Object
  weka.core.converters.ConverterUtils.DataSource
```

**All Implemented Interfaces:**: java.io.Serializable, RevisionHandler

**Enclosing class:**: ConverterUtils

---

``` public static class ConverterUtils.DataSource extends java.lang.Object implements java.io.Serializable, RevisionHandler ```

Helper class for loading data from files and URLs. Via the ConverterUtils
class it determines which converter to use for loading the data into
memory. If the chosen converter is an incremental one, then the data
will be loaded incrementally, otherwise as batch. In both cases the
same interface will be used (`hasMoreElements`,
`nextElement`). Before the
data can be read again, one has to call the `reset` method.
The data source can also be initialized with an Instances object, in
order to provide a unified interface to files and already loaded datasets.

**Version:**
:   $Revision: 6416 $

**Author:**
:   FracPete (fracpete at waikato dot ac dot nz)

**See Also:**: `hasMoreElements(Instances)`, `nextElement(Instances)`, `reset()`, `ConverterUtils.DataSink`, Serialized Form

---

**Constructor Summary**

`ConverterUtils.DataSource(java.io.InputStream stream)`
  
          Initializes the datasource with the given input stream.

`ConverterUtils.DataSource(Instances inst)`
  
          Initializes the datasource with the given dataset.

`ConverterUtils.DataSource(Loader loader)`
  
          Initializes the datasource with the given Loader.

`ConverterUtils.DataSource(java.lang.String location)`
  
          Tries to load the data from the file.


| **Method Summary** | |
| --- | --- |
| `Instances` | `getDataSet()`             returns the full dataset, can be null in case of an error. |
| `Instances` | `getDataSet(int classIndex)`             returns the full dataset with the specified class index set, can be null in case of an error. |
| `Loader` | `getLoader()`             returns the determined loader, null if the DataSource was initialized with data alone and not a file/URL. |
| `java.lang.String` | `getRevision()`             Returns the revision string. |
| `Instances` | `getStructure()`             returns the structure of the data. |
| `Instances` | `getStructure(int classIndex)`             returns the structure of the data, with the defined class index. |
| `boolean` | `hasMoreElements(Instances structure)`             returns whether there are more Instance objects in the data. |
| `static boolean` | `isArff(java.lang.String location)`             returns whether the extension of the location is likely to be of ARFF format, i.e., ending in ".arff" or ".arff.gz" (case-insensitive). |
| `boolean` | `isIncremental()`             returns whether the loader is an incremental one. |
| `static void` | `main(java.lang.String[] args)`             for testing only - takes a data file as input. |
| `Instance` | `nextElement(Instances dataset)`             returns the next element and sets the specified dataset, null if none available. |
| `static Instances` | `read(java.io.InputStream stream)`             convencience method for loading a dataset in batch mode from a stream. |
| `static Instances` | `read(Loader loader)`             convencience method for loading a dataset in batch mode. |
| `static Instances` | `read(java.lang.String location)`             convencience method for loading a dataset in batch mode. |
| `void` | `reset()`             resets the loader. |

**Methods inherited from class java.lang.Object**

`equals, getClass, hashCode, notify, notifyAll, toString, wait, wait, wait`

**Constructor Detail**

### ConverterUtils.DataSource

```
public ConverterUtils.DataSource(java.lang.String location)
                          throws java.lang.Exception
```

:   Tries to load the data from the file. Can be either a regular file or
    a web location (http://, https://, ftp:// or file://).

    **Parameters:**: `location` - the name of the file to load **Throws:**: `java.lang.Exception` - if initialization fails

---


### ConverterUtils.DataSource

```
public ConverterUtils.DataSource(Instances inst)
```

:   Initializes the datasource with the given dataset.

    **Parameters:**: `inst` - the dataset to use

---


### ConverterUtils.DataSource

```
public ConverterUtils.DataSource(Loader loader)
```

:   Initializes the datasource with the given Loader.

    **Parameters:**: `loader` - the Loader to use

---


### ConverterUtils.DataSource

```
public ConverterUtils.DataSource(java.io.InputStream stream)
```

:   Initializes the datasource with the given input stream. This stream
    is always interpreted as ARFF.

    **Parameters:**: `stream` - the stream to use


**Method Detail**

### isArff

```
public static boolean isArff(java.lang.String location)
```

:   returns whether the extension of the location is likely to be of ARFF
    format, i.e., ending in ".arff" or ".arff.gz" (case-insensitive).

    :   **Parameters:**: `location` - the file location to check **Returns:**: true if the location seems to be of ARFF format

---


### isIncremental

```
public boolean isIncremental()
```

:   returns whether the loader is an incremental one.

    :   **Returns:**: true if the loader is a true incremental one

---


### getLoader

```
public Loader getLoader()
```

:   returns the determined loader, null if the DataSource was initialized
    with data alone and not a file/URL.

    :   **Returns:**: the loader used for retrieving the data

---


### getDataSet

```
public Instances getDataSet()
                     throws java.lang.Exception
```

:   returns the full dataset, can be null in case of an error.

    :   **Returns:**: the full dataset **Throws:**: `java.lang.Exception` - if resetting of loader fails

---


### getDataSet

```
public Instances getDataSet(int classIndex)
                     throws java.lang.Exception
```

:   returns the full dataset with the specified class index set,
    can be null in case of an error.

    :   **Parameters:**: `classIndex` - the class index for the dataset **Returns:**: the full dataset **Throws:**: `java.lang.Exception` - if resetting of loader fails

---


### reset

```
public void reset()
           throws java.lang.Exception
```

:   resets the loader.

    :   **Throws:**: `java.lang.Exception` - if resetting fails

---


### getStructure

```
public Instances getStructure()
                       throws java.lang.Exception
```

:   returns the structure of the data.

    :   **Returns:**: the structure of the data **Throws:**: `java.lang.Exception` - if something goes wrong

---


### getStructure

```
public Instances getStructure(int classIndex)
                       throws java.lang.Exception
```

:   returns the structure of the data, with the defined class index.

    :   **Parameters:**: `classIndex` - the class index for the dataset **Returns:**: the structure of the data **Throws:**: `java.lang.Exception` - if something goes wrong

---


### hasMoreElements

```
public boolean hasMoreElements(Instances structure)
```

:   returns whether there are more Instance objects in the data.

    :   **Parameters:**: `structure` - the structure of the dataset **Returns:**: true if there are more Instance objects available **See Also:**: `nextElement(Instances)`

---


### nextElement

```
public Instance nextElement(Instances dataset)
```

:   returns the next element and sets the specified dataset, null if
    none available.

    :   **Parameters:**: `dataset` - the dataset to set for the instance **Returns:**: the next Instance

---


### read

```
public static Instances read(java.lang.String location)
                      throws java.lang.Exception
```

:   convencience method for loading a dataset in batch mode.

    :   **Parameters:**: `location` - the dataset to load **Returns:**: the dataset **Throws:**: `java.lang.Exception` - if loading fails

---


### read

```
public static Instances read(java.io.InputStream stream)
                      throws java.lang.Exception
```

:   convencience method for loading a dataset in batch mode from a stream.

    :   **Parameters:**: `stream` - the stream to load the dataset from **Returns:**: the dataset **Throws:**: `java.lang.Exception` - if loading fails

---


### read

```
public static Instances read(Loader loader)
                      throws java.lang.Exception
```

:   convencience method for loading a dataset in batch mode.

    :   **Parameters:**: `loader` - the loader to get the dataset from **Returns:**: the dataset **Throws:**: `java.lang.Exception` - if loading fails

---


### main

```
public static void main(java.lang.String[] args)
                 throws java.lang.Exception
```

:   for testing only - takes a data file as input.

    :   **Parameters:**: `args` - the commandline arguments **Throws:**: `java.lang.Exception` - if something goes wrong

---


### getRevision

```
public java.lang.String getRevision()
```

:   Returns the revision string.

    :   **Specified by:**: `getRevision` in interface `RevisionHandler`
    :   **Returns:**: the revision


---


|  |  |  |
| --- | --- | --- |
| **Overview** \| **Package** \| **Class** \| **Tree** \| **Deprecated** \| **Index** \| **Help** \| **Weka's home** | |  |
| **PREV CLASS**   **NEXT CLASS** | **FRAMES**    **NO FRAMES**     **All Classes** |  |
| SUMMARY: NESTED \| FIELD \| CONSTR \| METHOD | DETAIL: FIELD \| CONSTR \| METHOD |  |


---
